# Awareness of Noise-Induced Hearing Loss and Use of Hearing Protection among Young Adults in Jordan

**DOI:** 10.3390/ijerph16162961

**Published:** 2019-08-17

**Authors:** Nasim Alnuman, Talha Ghnimat

**Affiliations:** Department of Biomedical Engineering, School of Applied Medical Sciences, The German-Jordanian University, Amman 11180, Jordan

**Keywords:** noise-induced hearing loss, attitude to noise, young adults, leisure noise, Jordan, hearing protection

## Abstract

Action to prevent noise-induced hearing loss is necessary, especially because many causes of permanent hearing loss are preventable. The aim of this study is to identify and raise awareness of the effect of loud sounds on hearing and effective ways to protect ears among young adults in Jordan. Using non-probability and convenience sampling, 245 students (113 female and 132 males, aged 21.5 years ± 2.18) from three universities participated in the study and filled the questionnaire completely. The questionnaire consisted of 19 questions targeting hearing health. The answers were analyzed statistically, and comparisons were made using the t-test. Hearing loss was regarded as an important issue by 64.1% of the participants. Among the participants, 58% already suffered from at least one hearing symptom, even though only 9.8% used earplugs to protect their hearing. After receiving information on noise-induced hearing loss, 56.3% were likely or somewhat likely to use earplugs in the future (*p* < 0.01). This indicates that education and knowledge have a strong influence on student attitudes. It is obvious that the awareness of noise-induced hearing loss among adults in Jordan is very low. The authorities, policymakers, media, and educational institutes should recognize their responsibility in raising the awareness of the danger of loud music among young adults.

## 1. Introduction

Hearing impairment is a major challenge for public health organizations. According to the World Health Organization (WHO), there are approximately 466 million people living with disabling hearing loss, including approximately 34 million children. Furthermore, of these, nearly 90% live in middle- and low-income countries. Previous reports have also highlighted the significance of noise-induced hearing loss (NIHL), both work- and recreational activity-related NIHL [1,2]. In the United States, the estimated percentage of individuals with hearing impairment is around 14.4% of adults aged 18 years and above, and approximately 10 million of them suffer from hearing loss due to noise exposure [3,4]. In the UK, approximately 11 million people have a hearing impairment [5].

Noise and loud sounds are reported to be an important factor leading to hearing loss. NIHL is reported to be the second main cause of sensorineural hearing loss, after age-related hearing loss, even though it is virtually entirely preventable [6]. Olusanya et al. outlined the primary and secondary activities for the prevention of disabling hearing impairment. These included avoidance of exposure to prolonged and excessive noise combined with education and enforceable rules [7]. Phillips et al. reported a 45% prevalence of NIHL within a study on 329 student musicians. They also noticed a bilateral notching within 11.5% of the musicians [8].

In a study on strongly amplified music, Meyer-Bisch found that young adults using personal cassette players for prolonged periods (>7 h/week) had a significantly shifted hearing threshold in comparison to subjects using the players for 2–7 h/week [9]. Dalton et al. also reported a significant increase in the likelihood of hearing loss with participation in noisy leisure activities that involve sound levels greater than 90 dBA. The study was based on a population of 3571 participants and a reduction greater than 25 dB was referred to as hearing loss in either ear or both [10]. Similar results were found in China, relating the hearing loss to two main factors, aminoglycoside antibiotics and noise exposure [11].

Tinnitus was observed by many researchers as the most common disturbance after hearing loud noises and loud music. In a study by Bogoch et al., 84.7% of music concert attendees experienced tinnitus, which is an important hearing loss symptom [12]. In the work of Degeest, 80% had either transient or chronic tinnitus after exposure to leisure noise within a sample of 151 individuals [13]. However, a study reported that ear pain was the most frequent symptom (22.5%) in a sample of 2151 college students, followed by noise sensitivity in 20.8% of the participants. Temporary and permanent tinnitus were found in 17.4% of the sample [14].

Worldwide, some studies have been conducted on the public’s awareness of hearing health and hearing loss. The results of these studies varied from one country to another. Joubert et al. conducted a study in Limpopo province, South Africa, showing that 89% of their sample (n = 297) acknowledged the relation between loud noise and hearing damage [15]. Chung et al. [16] studied awareness on hearing loss using a web-based survey, showing that only 8% of participants consider hearing loss to be a big problem, which was a low priority in comparison with other health issues like depression and sexually transmitted diseases, which were considered by 44% and 50% of respondents, respectively, to be very big problems. They further reported earplug use by only 14% of participants. However, after reading about NIHL as a potential permanent health issue from loud music, 66% of participants intended to use earplugs in the future. In another study from the same researcher group five years later, the percentage of participants who viewed hearing loss as a big problem increased to 30%, and they concluded that education on hearing loss can lead to increased opportunities for protecting the hearing of adults [17].

In a study by Balanay et al. [14] conducted in 2015 on the attitudes toward noise among college students, 41% (n = 2151) reported using hearing protection during noisy activity. This use of hearing protection was associated with having hearing symptoms. For male participants, sports activities were the most important events with loud sounds (75.1%), followed by discos (51.9). For female participants, sporting events were the second most important noisy activity (51.8%) and the first was discos (57.3%).

More frequently, young adults are hearing loud music for long hours and exposing themselves to loud music in concerts and clubs. Sound levels of 120 to 140 dB have been reported in rock concerts [18], and average noise levels greater than 100 dB in nightclubs and pop concerts [19]. Many regulations exist to protect adult workers in the workplace from excessive noise exposure and from the risks of NIHL [16]. On the other hand, there is a lack of regulations and guidelines for non-occupational conditions. Vogel et al. concluded that authorities and the music industry must take responsibility to protect youth from loud music on MP3 players. The conclusions were based on a Delphi study with 30 experts from different fields including education, community health professions, scientific research, music entertainment, education, and others [20].

To our knowledge, there is no study on the awareness of NIHL among young people in Jordan and nearby countries. In this study, the authors use the results of a survey to evaluate the knowledge of young adults of hearing health and the issues related to NIHL. It includes an evaluation of hearing symptoms related to noise exposure, the acceptance of students to use ear protection, and the duration of exposure to loud music. The study tries to build a baseline of knowledge that may be used in any awareness-raising campaign and tries to better understand young adult’s acceptance to using hearing protection devices.

## 2. Methods

The research was conducted in three universities (two public universities and one private university) in Jordan in the fall semester of 2018. The population of Jordan is 9.5 million citizens (2015). Jordan has 10 public and 16 private universities. A non-probability, convenience sampling strategy was used in selecting the participants.

A total of 287 individuals were invited to participate in the questionnaire. Out of them, 250 participants accepted the invitation. A total of 245 participants (age = 21.52 ± 2.18 years) filled the questionnaire out completely. Only 5 participants did not fill it completely, and their forms are excluded from the study; 46.1% (n = 113) were females and 53.9% (n = 132) were males (Table 1). Their ages ranged from 17 years to 27 years. All participants were undergraduate or graduate students.

The questionnaire used in this study was based on the 28-question survey from the Massachusetts Eye and Ear Infirmary, the Harvard School of Public Health, and Cogent Research, Inc. [16]. The original survey contained questions about health issues other than hearing.

The final questionnaire consisted of five demographic items about age, gender, educational level, place of residence, and family income. A total of 18 open- and close-ended questions on attitude toward hearing loss, hearing problem symptoms, NIHL, noise and loud sound exposure duration, the use of hearing protection, mainly earplugs, and the perception of the adults toward using them, were included. The full questionnaire can be found in Appendix A.

A pilot study was done prior to the data collection to ensure clarity of the questions, content, and face validity. The pilot study was done on 10 participants and their comments on the questions were collected and considered in rewriting the questionnaire. Their questionnaires were later excluded from the final data analysis. After the pilot study, the questions were reviewed and reformatted.

Students from the three universities were selected by asking every fifth student passing by a student’s gathering area to fill the questionnaire. Participants were fully informed about the study verbally and assured of their rights not to complete the questionnaire anytime with no consequences. The questionnaire was self-administrated while one of the researchers was present during the administration. All filled surveys were verified for completeness during data cleaning and incomplete questionnaires (n = 5) were discarded.

The research was conducted in accordance with the principles of the Declaration of Helsinki. The participants received no rewards for their participation. Permissions were gathered from all participating universities before conducting the research on the campus. Forms were approved by the Deanship of Academic Research at the German-Jordanian University.

Data were stored on an Excel spreadsheet, and then transferred to the commercial program MATLAB (R2018b, academic use) and analyzed using the statistical toolbox. The frequencies of occurrence and percentages are given for categorical questions. The mean values and ranges are given for continues measures. Statistical comparisons between values are provided where applicable using the two-tailed N-1 Chi-Square test for different groups and McNemar’s test for paired data. *p* < 0.05 was used for a statistically significant difference.

## 3. Results

The results are presented in the same flow of the questions in the survey. The first part aimed to identify the attitude toward hearing loss. Within the selected sample, 64.1% (n = 157) believed that hearing loss is a big or very big problem for people suffering from it. On the other hand, 35.9% (n = 88) viewed it as either no problem at all or not that big of a problem. In comparison with depression as another important health issue for young adults, 76.3% (n = 187) felt that depression is a big or very big problem (this percentage was 12.2% higher than hearing loss, which was significant (x^2^ = 10.01, *p* < 0.01, *p* = 0.0016)), and only 23.7% felt that it is either not a big problem or not a problem at all.

When asked about imagining themselves suffering from hearing loss, there was a slight insignificant change in their opinion, such that 63.3% assumed that hearing loss would be a challenging problem if they were suffering from it. However, the percentage of those believing depression is a big or very big problem if they were suffering from it dropped significantly to 63.7% from 76.3% (x^2^ = 9.68, *p* < 0.01, *p* = 0.002).

Across all participants, 65.7% (n = 161) already have some idea or have heard something about hearing loss. For those 161 participants, the primary source of their knowledge varied from media, TV shows, and the internet for 46.0% (n = 74), followed by 31.7% who experienced one or more forms of temporary hearing loss themselves after participating in an activity that included loud sounds, hearing loud music, ear infection, or fireworks. The remaining 22.4% experienced hearing loss through relatives (mainly grandparents) or friends suffering from hearing loss problems. Around one-third of participants (34.3%, n = 84) have never faced or heard anything related to hearing loss before reading the questionnaire.

**Figure 1:**
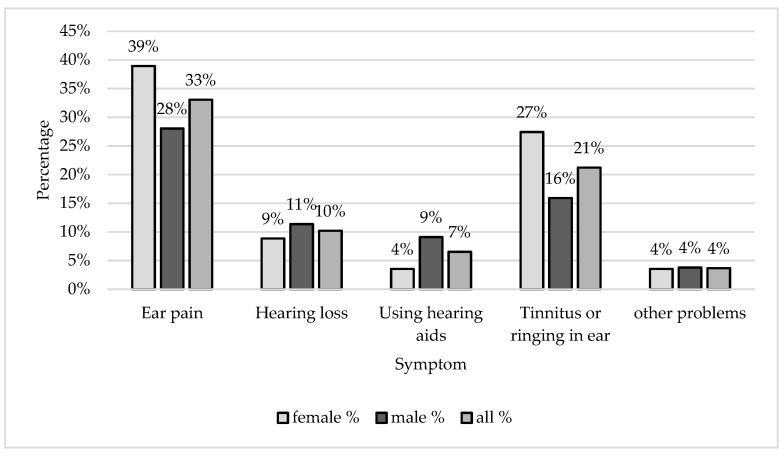
The frequency of different hearing symptoms experienced by the male and female participants. The percentages are calculated in reference to the whole sample size.

In the second part, the participants were asked if they experienced any noise-related hearing symptoms based on their own judgment. Within the male participants, 53% reported that they had at least one symptom. For female participants, the percentage was 62.8%, and this percentage was not significantly different from that of males (x^2^ = 2.38, *p* = 0.123). The overall percentage for both genders was 57.6%. Figure 1 presents the different hearing symptoms related to noise exposure. Ear pain was reported as the most common (33.1%), and the second hearing symptom was tinnitus 21.22%. Comparing the results for males and females, the percentage of females that experienced tinnitus (27.4%) was significantly higher (x^2^ = 4.80, *p* = 0.0285) than for males (15.9%). However, the differences in ear pain between males (28.0%) and females (38.9%) was not statistically significant (x^2^ = 3.26, *p* = 0.071).

The students aged 21 years old had the highest percentage of at least one hearing symptom (64.6%). However, within the relatively small sample size, there was no clear statistically significant difference between the different ages and frequency of hearing symptoms.

The next part of the questionnaire examined the relation between family income and the existence of hearing symptoms. The percentage of students who experienced at least one hearing symptom among families with an above-average income (57.6%) was significantly higher (x^2^ = 5.96, *p* = 0.0146) than those belonging to families with average income (33.3%), and significantly higher (x^2^ = 5.78, *p* = 0.01162) than those belonging to families with a below-average income (22.2%). There was no significant difference between middle- and low-income students (x^2^ = 0.853, *p* = 0.356).

In the third part of the questionnaire, the participants were asked if they participated in a list of activities that included loud sounds, and how often they do that. Visiting parties with loud music and listening to loud music with headsets and other devices were reported as the most common activities, with 79.2% and 81.2%, respectively. The different percentages are shown for both males and females in Table 2. There is strong evidence that females participated more than males in weddings and parties where loud music is played (*p* < 0.01). However, males’ attendance at clubs, raves, and discos (49.2%) was significantly higher than females (15.0%) with a *p* < 0.01. The overall percentage of students exposed to loud music in at least one of these activities is 90.6%, and the rest (9.4%) do not participate in any of these loud activities. Regarding these activities, it is important to highlight that 60.6% listen to loud music using media devices on a daily basis.

The percentage of students who experienced at least one hearing symptom often or very often after participating in activities involving loud sounds is 72.3% after participating in concerts, 65.9% after attending discos and raves, 53.6% after weddings and parties with loud music, and the smallest percentage was for personal music players and media devices with 43.2%.

### The Use of Hearing Protection

To evaluate attitudes toward the use of hearing protection, the students were asked about their opinions regarding the use of hearing protection by others and how they accept it. The opinions were grouped into four categories: 44.1% viewed it as funny and cool; 30.2% viewed earplugs negatively; as ugly and weird; 23.67% thought it was normal and a part of personal freedom; and only 2% saw it positively, and as part of having a good attitude toward protecting their hearing. A comparison based on gender gave no statistically significant difference (x^2^ = 0.065, *p* = 0.7992) when considering the opinion “it is weird and ugly” (31.0% for females, 29.5% for males). Similarly, there was no significant difference between genders of who saw the use of earplugs as normal (26.5% for females, 21.2% for males, x^2^ = 0.943, *p* = 0.3314). However, a significantly higher percentage of females (53.1%) saw it as funny and cool in comparison with males (36.4%, x^2^ = 6.58, *p* = 0.0103).

The reported use of protection for hearing was just 9.8% (n = 24) of the whole sample size, which was divided into 11 participants wearing it to protect their hearing, 6 because they already had hearing problems, 5 to avoid sleeping problems after loud music, and 2 because they got nervous hearing loud music. From the participants who experienced at least one hearing symptom, 12.8% already used hearing protection, and only 5.8% of those with no hearing symptoms used hearing protection. This difference was not statistically significant (X^2^ = 3.31, *p* = 0.0691). As the number of hearing protection users was very small, comparison based on gender or hearing problems is not indicative. Out of the 90.2% that have never used ear protection, 43.0% believe there is no need to use them and prefer loud sounds, 14.9% never heard about hearing protection before, and 9.0% do not want to use it due to its shape. Within this question, 73 participants did not write the reason for not using ear protection (33.0%).

**Figure 2:**
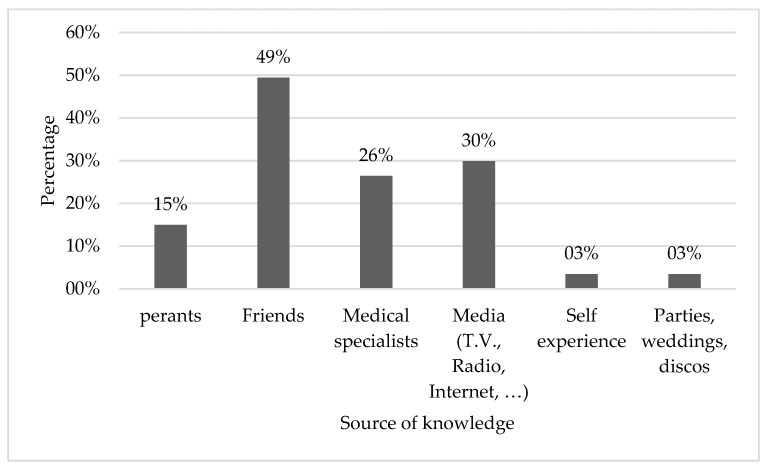
The sources of knowledge about hearing protection and earplug use.

Even though only 9.8% of the participants already used hearing protection, 35.5% (n = 87) of the participants had heard about the use of earplugs. The source of knowledge was mainly friends followed by media (TV, internet, radio, etc.). Figure 2 shows the different sources of knowledge about the importance of using ear protection, specifically earplugs.

When asked about their knowledge of induced hearing loss, 81.2% (n = 199) admitted having known that loud sounds can be damaging to the hearing, and just 18.8% had no idea before reading this survey. There was no significant difference between males and females (x^2^ = 0.159, *p* = 0.69).

**Figure 3:**
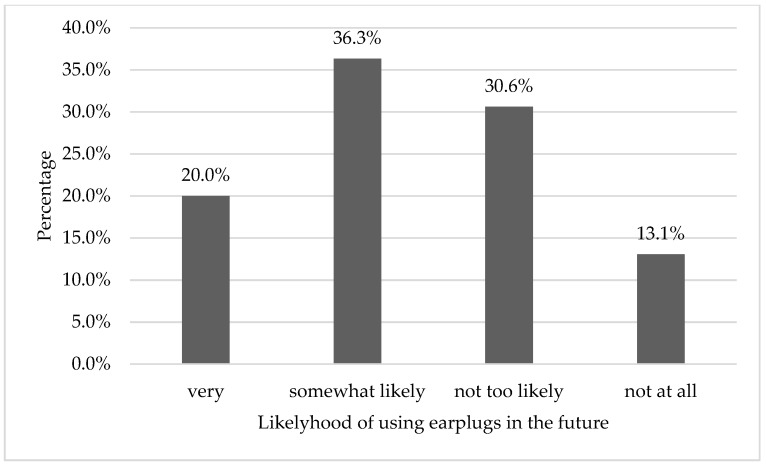
Likelihood of using earplugs and ear protection in the future.

After reading this survey and the information paragraph, 56.3% expressed that they will very likely or somewhat likely use earplugs in the future. In comparison with the 9.8% of participants who used earplugs. This change is statistically significant and strongly indicates a change in attitudes (paired x^2^ = 103.0, *p* < 0.01). There was 134 (54.7%) participants who did not know where to get earplugs. Figure 3 shows the percentages of the likelihood of using earplugs and ear protection.

**Figure 4:**
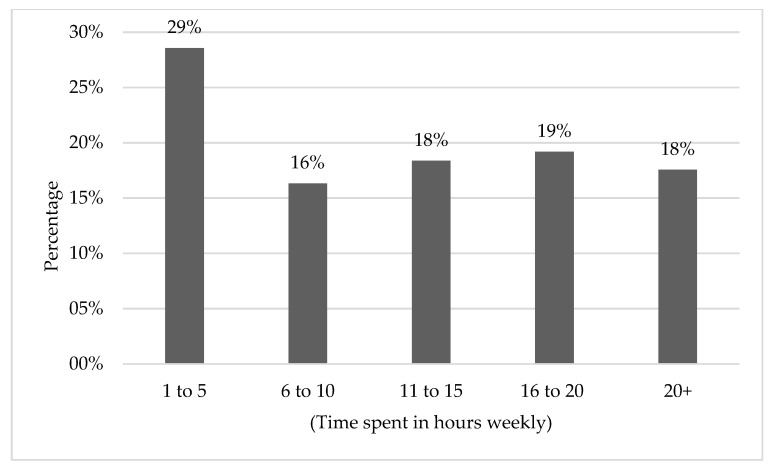
The time spent listening to music and using audio devices weekly.

The students were asked about the time they spend hearing music weekly. Figure 4 shows the percentage of students in each time category. There was no correlation between the time spent listening to music weekly and the existence of at least one hearing symptom (x^2^ = 6.3, *p* = 0.178). However, the presence of tinnitus showed a statistically significant correlation with the time spent listening to music weekly (x^2^ = 12.4, *p* = 0.015). No significant correlation was found between ear pain and music listening time (x^2^ = 5.87, *p* = 0.21). For listening to music, 26.9% use a headset, 20% use loudspeakers, and 49.8 use both, while 3.3% do not listen to music.

## 4. Discussion

The current survey provided insight into the awareness and knowledge of hearing loss among young adults. Almost two-thirds of the sample believed that hearing loss is a big problem or a very big problem, but they believed it is less important than depression. Similarly, in the survey conducted by Chung et al., the participants viewed depression as a more important problem. In this study, 27.8%, as compared to 8% in the Chung sample, considered it to be a very big problem. The same attitude was noticed in the Quintanilla-Dieck et al. study, where the percentage was around 31%. Furthermore, the differences between males and females were not statistically significant, which was reported in the other studies [16,17].

The percentage of participants who already experienced something related to hearing loss was 65.7%, which is relatively high in comparison with other studies [16,17], (*p* < 0.01). However, this study was performed on graduate and undergraduate students, while the other studies included younger high school students.

The percentage of participants who experienced at least one symptom of hearing loss was 53.0%, which was similar to the study of Quintanilla-Dieck et al. [17], and higher than the reported results in the work of Balanay and Kearney [14]. All three studies showed no significant difference between males and females. The reported hearing symptoms in relation to family income showed a significant correlation between high income and hearing symptoms. This may be explained by the ability of students from high-income families to participate more in free time activities in general, which includes noisy activities.

It is noticed that of those with at least one hearing symptom, 65.9% attended discos and nightclubs. Johnson et al. also reported high percentages after visiting nightclubs [21], where 88.3% of their sample had tinnitus and 66.2% had impaired hearing the day after visiting nightclubs. The most common hearing symptom found in this study was ear pain followed by tinnitus, which is similar to Balanay and Kearney’s results [14]. In [16,17], tinnitus was reported as the most common hearing symptom.

Of the type of activities that include loud sounds, using headsets and other media devices (81.2%) was the most common, followed by visiting parties and wedding celebrations (79.2%), with a small difference between them. Lass et al. [22] reported dances as the most common, with 69.9%, which is considered within the same field. Balanay reported sports events (59.7%) as the most common [14]. Joubert et al. reported music in taxis as the most common [15]. It is clear that there are differences between the different studies, which we believe are due to cultural differences. There are no studies, to our knowledge, that have surveyed the student dominant activities between different countries, and more investigation is needed in the future to better understand the variabilities.

In this study, it was also clear that the number of male students attending discos and clubs was higher than female students in Jordan, in contrast to a study in the USA [14] that showed that more females attend discos than males.

The overall use of ear protection was very low in this study (9.8%), even though 57.6% had at least one hearing symptom, and furthermore, 35.5% already knew about earplugs and their uses. This was similar to what was found in previous studies [14,16,17,23,24].

A majority of the participants admitted to knowing about the relation between loud sounds and hearing loss. Similarly, Joubert reported that 89% (n = 265) of their sample admitted to knowing about the relation between loud noise and hearing problems, and Quintanilla-Dieck et al. reported more than two-thirds of their sample (n = 2500) knew about noise-induced hearing loss [17].

The results on attitudes toward the use of hearing protection and the specific result that most respondents knew about the relation between loud noise and hearing loss needs, in our opinion, more studies and analysis in the future on larger samples and with consideration of psychosocial factors. According to this survey, only 2% (n = 5) viewed using earplugs positively and for the right reason of protecting the ears, and 30.2% responded negatively, viewing earplugs as either ugly or weird. Other researchers, like [16], also reported the negative response, with 41% viewing earplugs negatively.

The results of the survey reveal a very strong opportunity for education to improve the attitude towards using earplugs and hearing protection. After education, 56.3% of participants were either very likely (20.0%) or somewhat likely (36.3%) to use protection in the future, which is a significant jump from the 9.8% that already use earplugs. Similarly, the MTV survey of 2002 and 2007 showed an opportunity to educate, where the percentage of participants increased from 14% using earplugs to 22% willing to use earplugs for the 2002 study, and 15% using earplugs to 28% willing to use them for the 2007 survey [16,17].

This study reveals a strong correlation between tinnitus and time spent listening to music. This dependency was not significant for other ear diseases within this study. The 2007 MTV survey also found that music listening activities were related to temporary tinnitus. A significant percentage (18%) spend more than 20 h weekly listening to music, and 37% spent more than 16 h a week. These measures are very indicative and need to be considered in any educational campaign in the future. Furthermore, in our opinion, action from the local authorities to legally force electronic device manufacturers and suppliers to have safety-warning labels on their devices, including information about NIHL, is needed.

The authors made every effort to avoid any bias in the study results. It is important to mention that bias may be present due to the size of the sample and that doing a study on a larger sample size and possibly in neighboring countries is recommended. Future studies may utilize audiometric tests to avoid any bias due to the self-reporting nature of this study.

The results indicate a clear need to raise the awareness of NIHL among young adults. This can be achieved primarily by focusing on advertising campaigns, utilizing the online media and university webpages. The authors believe that such campaigns, even if they are costly, can help improve society’s health and reduce the future cost of NIHL. For example, the estimated reduction in income for people with hearing loss in the UK is around 2000 pounds annually, which adds up to four billion annually over the whole UK [25]. The awareness and hearing education campaigns are not enough alone unless they are combined with reduction in sound level limits in concerts, discotheques, and personal media players [26]. Preventive and awareness campaigns lead to an increase in the use of hearing protection and increased the negative attitude toward noise and loud sounds [26,27]. More investigation is needed to study the long-term effects of hearing campaigns [27,28]. The authors did not find information about any hearing education campaigns in Jordan or the region. In other countries, many campaigns were held and utilized different media like newspapers, and the internet. These campaigns were effective in some situations leading to changes in the regulations related to noise and hearing protection, however, the long-term effect of these efforts has not been investigated. It is recommended to organize campaigns with limited goals and long duration, rather than short-lived campaigns with temporary effect [28,29]. We believe the Jordanian regulations, like many other known regulations worldwide, try to regulate the exposure to loud sounds in society, regulate the isolation of clubs and festival halls, and put limits on the use of loud speakers in open areas [30]. However, there is a weakness in the implementation and follow-up of these regulations, which is, in our opinion, due the lack of awareness about the effects of loud sounds on health and hearing, and the lack of knowledge that this may lead to NIHL in the long term.

## 5. Conclusions

The available data on NIHL and the attitudes toward hearing loss and hearing protection among university students is limited. Some studies have been performed in developed high-income countries. However, less has been done in middle- and low-income countries. We believe this study adds to the already existing studies worldwide and highlights the issue in one of the Middle Eastern countries. Even though the sample size, taken from three different universities, is relatively small (n = 245), it can be seen as guidance for more studies and for policymakers. The results of this study show the need for action by the relevant authorities and institutes to enhance public knowledge on NIHL. NIHL can be controlled and prevented by taking proper actions as suggested by Olusanya et al. [7]. More TV programs and media sources should allocate space for this important issue. Fast action can save the country money in the near and distant future because of the high costs of services for hearing impaired individuals, who may appear in the late adulthood.

Even though knowledge of hearing loss and the existence of ear protection is comparable with other studies conducted in other countries. There is a clear lack of knowledge about the importance of using earplugs and hearing protection. This study indicated potential for education within the young adult segment of the population.

## Figures and Tables

**Table 1:** Participants’ demographic data.

Characteristic	n	Percentage %
Gender		
Male	132	53.9
Female	113	46.1
Age		
17–19	47	19.2
20–22	119	48.6
23 years and above	79	32.2
Educational level		
Undergraduate	207	84.5
Graduate	38	15.5
Family Income		
Above Average	33	13.5
Average	93	38.0
Below average	18	7.3
Don’t know	60	24.5
Prefer not to answer	41	16.7

**Table 2:** Distribution of the students’ participation in the different noisy activities.

Noisy Activity	Male (n = 132)		Female (n = 113)		*p* Value	x^2^
	n	%	n	%		
Listening to loud music on a stereo/headset	103	78.0%	96	85.0%	0.163	1.95
Clubs, raves, and discos	65	49.2%	17	15.0%	<0.01	31.87
Concerts	76	57.6%	54	47.8%	0.126	2.34
Weddings and parties (with loud music)	93	70.5%	101	89.4%	<0.01	13.16

Note: The sum of the percentages exceeds 100% because many of them act in more than one activity.

## Appendix A

The questionnaire used in this study. Modified from The Massachusetts Eye and Ear Infirmary, the Harvard School of Public Health, and Cogent Research, Inc. [16].

**A Questionnaire for Evaluating Noise Induced Hearing Loss and People Awareness about the Effects of Loud Sounds on Hearing and Health:**

Participant no: ____________    Date: ___________________

**(Please write X in front of the proper answer)**

Age: ________________       Gender: __Male __ Female

Educational status: __________________

Country of residence: __________________

**1- These are two problems facing young people these days? Please determine how big do you feel these problems, even if they do not affect you personally?**

	A very Big Problem	A Big Problem	Not that Big	Not at All
Hearing loss or hearing problems				
Depression				

**2- Now, could you please imagine yourself affected by one of these issues, how will you evaluate these problems**

	A very Big Problem	A Big Problem	Not that Big	Not at All
Hearing loss or hearing problems				
Depression				

● In this part of questions, we will point to the hearing problems:

**3- Have you ever faced, read, heard or seen anything which is related to hearing loss problems?**

__ a. Yes

__ b. No (if no go to question 5)

**4- How/Where have you faced, heard, read or seen something about hearing Loss?**

_______________________________________________

_______________________________________________

**5- Have you ever experienced or ever been affected by any type of hearing Problems, such as:**

__ a. Ear pain (Ex. due noise exposure)

__ b. Temporary Hearing Loss/Noise Sensitivity

__ c. Already use/used hearing aids

__ d. Tinnitus or Ringing in Ear (is a condition in which there is a sense of sound within the ear without the existence of any external sound)

__ e. Any other problems that are not listed: ___________________

__ f. None of the a above

**6- In the following schedule there are some activities that may cause hearing related problems; do you frequently attend such activities?**

	Yes	No	How many times in a week/year?
Listening to loud music on a stereo/headset, others			
A club, rave and discos			
Concerts			
weddings and parties (with loud music)			

**7- After or during which one of these activities have you experienced a hearing related problem?**

	Very often	Often	Not often	Never
Listening to loud music on a stereo/headset				
During or after going to a disco, club, …				
During or after going to a concert				
During or after going to weddings and parties (with loud music)				

**Please read the following paragraph:**

**An earplug is a device that is inserted in the ear canal to protect the wearer’s ears from loud sounds and noise**

**8- What do you feel, when you see someone wearing an ear plug during a party, wedding, disco, …? (Funny, cool, ugly, healthy, …………)**

___________________________________________________________________

**9- Have you ever worn earplugs at a rave, club, concerts, weddings or any other places where loud music was being played?**

__ a. Yes (If yes, go to question 11)

__ b. No (If No, go to question 10)

**10- Could you please justify, why you did not follow ear protection devices consideration?**

__________________________________________________________________

__________________________________________________________________

**11- Why you have planned to wear Earplugs?**

__ a. Since loud music cause hearing problems (I wear to protect my hearing system)

__ b. Since loud music is simply annoying (make me nervous)

__ c. Since I have already hearing problems

__ d. After hearing loud music I have problems in sleeping

__ e. Others________________________________________________________

*** Please read the following paragraph:**

**Loud sounds affect the hearing sense and hearing loud music for a long time will increase the probability of hearing loss and hearing related problems such as (sleeplessness). Using earplugs (Hearing protection system devises) can reduce 75% of related sound. And that protect your hearing and it is already recommended to be used.**

*** Now could you please answer the following questions.**

**12- Have you ever met any person or seen any program that suggested wearing earplugs in such parties before reading this paragraph?**

__ a. Yes

__ b. No

**13- Where have you got this information?**

__ a. Parents

__ b. Friends

__ c. Doctors, Nurses

__ d. From media (TV, Internet, Radio, … etc.)

__ e. since I experienced a problem (It is my first time to experience that)

__ f. Heard about it at a disco, weddings, parties, … etc.

__ g. Others ________________________

**14- Before reading the previous Have you ever known that loud music and noise could damage the hearing system?**

__ a. Yes

__ b. No

**15- After this information, how likely you are to use the Earplugs next time that you go to a party, rave, concert or wedding?**

__ a. Very likely

__ b. Somewhat likely

__ c. Not too likely

__ d. Not at all, why? (____________________________________________________)

**16- Do you have any idea, from where can you get an earplug (Hearing protection devise)?**

__ a. Yes

__ b. No

(If no, read the following suggestions: in a pharmacy, health clinic, at your doctor office, at a hearing aid store or at an audiologist’s office)

**17- In a typical week, how much time do you spend on hearing music?**

__ a. 1–5 h

__ b. 6–10 h

__ c. 11–15 h

__ d. 16–20 h

__ e. More than 20 h

**18- To hear music what do you use Mostly?**

__ a. Mainly Headset

__ b. Mainly from Loudspeakers or TV

__ c. Both (Almost equivalent)

**19- To the best of your knowledge, how much was your family income in the last year?**

__ a. above the average

__ b. below the average

__ c. around the average

__ d. Have no idea

__ e. Prefer not to answer

               **Thank you for your help**
